# Benefit of neoadjuvant immunotherapy plus chemotherapy in locally advanced esophageal squamous cell carcinoma: trial sequential analysis

**DOI:** 10.1097/JS9.0000000000001660

**Published:** 2024-05-20

**Authors:** I-Wen Chen, Ting-Sian Yu, Kuo-Chuan Hung

**Affiliations:** a Department of Anesthesiology, Chi Mei Medical Center, Liouying; b Department of Anesthesiology, Chi Mei Medical Center, Tainan City; c Department of Anesthesiology, E-Da Hospital, I-Shou University, Kaohsiung City, Taiwan

*Dear Editor,*

We read with great interest the article by Xu *et al*.^1^ titled ‘Comparison of efficacy and safety between neoadjuvant chemotherapy (NCT) and neoadjuvant immune checkpoint inhibitors combined with chemotherapy (NICT) for locally advanced esophageal squamous cell carcinoma: a systematic review and meta-analysis.’ The authors should be congratulated on conducting this important meta-analysis comparing NICT versus NCT for locally advanced esophageal squamous cell carcinoma (ESCC). The meta-analysis found that NICT significantly improved pathologic complete response (pCR) and major pathologic response rates compared to NCT, with odds ratios of 4.24 (95% CI, 2.84–6.32) and 3.30 (95% CI, 2.31–4.71), respectively^1^. No significant differences were found in R0 resection rates, incidence of treatment-related adverse events, or postoperative complications between the two approaches. These findings suggest that NICT is a promising neoadjuvant strategy for locally advanced ESCC.

This topic is of great importance given the poor prognosis of locally advanced ESCC and the need for more effective neoadjuvant treatment options. While the significantly improved pCR rates with NICT are encouraging^1^, caution is advised when interpreting these results because of the limited sample sizes of the included studies. Additional analysis by performing a trial sequential analysis (TSA) may be informative for readers to assess further the evidence supporting the pCR findings. TSA is a statistical approach that combines information size calculation with an adjusted threshold for statistical significance in meta-analyses to control for the risk of random errors due to sparse data and repeated testing^2,3^. It can determine whether a meta-analysis has an adequate cumulative sample size to detect or reject a certain intervention effect.

As TSA was not performed in the original meta-analysis^1^, we performed this analysis using the raw data provided in the meta-analysis^1^. For TSA, we assumed an anticipated relative risk reduction of 20%, *α*=0.05, and *β*=0.20. TSA was conducted using TSA software (version 0.9.5.10 beta) (Copenhagen Trial Unit, Centre for Clinical Intervention Research, Rigshospitalet, Copenhagen, Denmark). The analysis revealed that the cumulative *Z*-curve crossed the required information size boundary (Fig. 1), indicating firm evidence of the benefits of pCR with NICT. This indicates that the significantly improved pCR rates with NICT are unlikely to be due to random errors caused by limited sample sizes.

**Figure 1:**
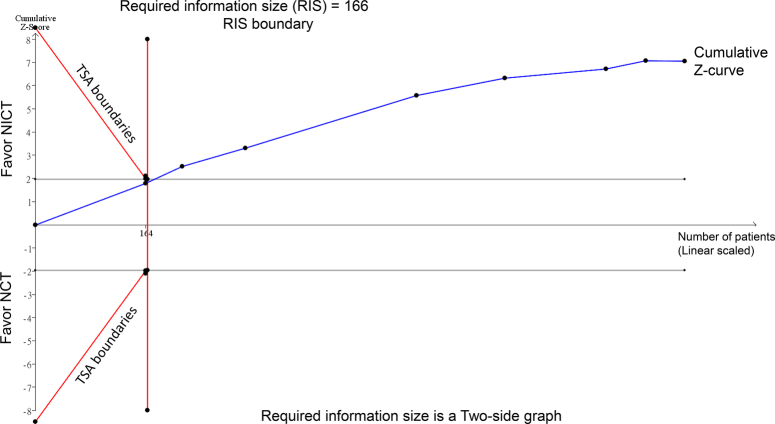
TSA assessing the efficacy of NICT versus NCT on pCR rates in locally advanced esophageal squamous cell carcinoma. The required information size of 166 patients was calculated based on an anticipated relative risk reduction of 20%, *α*=0.05, and *β*=0.20. The blue cumulative *Z*-curve crosses the required information size boundary, indicating firm evidence for the benefits of pCR with NICT. NCT, neoadjuvant chemotherapy; NICT neoadjuvant immune checkpoint inhibitors combined with chemotherapy; pCR, pathologic complete response; TSA, trial sequential analysis.

In conclusion, this original meta-analysis^1^ provides important insights into the potential of NICT as a neoadjuvant treatment for locally advanced ESCC. TSA supported the validity of the benefits of pCR. However, further high-quality randomized trials with adequate power and long-term survival data are needed to confirm these promising findings before changes to clinical practice can be recommended.

## Ethical approval

Not applicable.

## Consent

No external funding was received for this study.

## Sources of funding

Not applicable.

## Author contribution

I-W.C. and K.-C.H.: wrote the main manuscript text; T.-S.Y.: prepared Figure 1. All authors read and approved the final version of the manuscript.

## Conflicts interest disclosure

The authors declare no conflicts of interest.

## Research registration unique identifying number (UIN)

Not applicable.

## Guarantor

Kuo-Chuan Hung.

## Data availability statement

The datasets used and/or analyzed in the current study are available from the corresponding author upon reasonable request.

## Provenance and peer review

This paper was not invited.
